# Reproducibility of Mandibular Cortical Index Classification Among Dental Examiners and a Single Generative AI Platform: An Observer Agreement Study

**DOI:** 10.3390/clinpract16080142

**Published:** 2026-07-31

**Authors:** Keisuke Seki, Minori Kashima, Taiki Akiyama, Atsushi Kobayashi, Ko Dezawa, Yoshimasa Takeuchi, Mika Furuchi, Atsushi Kamimoto

**Affiliations:** 1 Department of Comprehensive Dentistry and Clinical Education, Nihon University School of Dentistry, Tokyo 101-8310, Japan; 2 Department of Oral and Maxillofacial Radiology, Nihon University School of Dentistry, Tokyo 101-8310, Japan

**Keywords:** osteoporosis screening, mandibular cortical index, dental panoramic radiography, inter-rater reliability, intra-rater reproducibility, generative AI, large language model, NotebookLM, kappa coefficient

## Abstract

**Background/Objectives:** The mandibular cortical index (MCI) is a valuable screening tool for osteoporosis on dental panoramic radiographs, but its assessment is subject to inter-examiner variability. This study evaluated the reproducibility and inter-rater agreement of MCI classification by a closed-source generative AI tool (NotebookLM, Google) compared with eight dentists of varying clinical experience. **Methods:** One hundred panoramic radiographs were classified according to the three-category MCI in two sessions held at least two weeks apart. Intra-examiner reliability, inter-examiner agreement, and agreement with a reference radiologist were assessed using linearly weighted kappa coefficients. The study was designed as a descriptive reliability study rather than a formal equivalence trial. **Results:** The intra-examiner reliability of the AI was exceptionally high (κ = 0.987). However, agreement between the AI and the dentists remained at “slight agreement” or lower (κ < 0.2) for every pairing, with 95% confidence intervals that included zero; no formal global hypothesis test was performed, and these individual interval estimates should not be interpreted as proof of the absence of agreement beyond chance. A “two-level discrepancy,” in which the AI interchanged Class 1 (normal) and Class 3 (severe), occurred in 10–18% of cases. The dentists showed a possible learning effect, with inter-examiner agreement improving between sessions. **Conclusions:** Despite the high reproducibility of the NotebookLM configuration evaluated in this study, agreement with the dentists remained at “slight” or lower (κ < 0.2) in MCI classification. As classifications were not validated against bone mineral density or an adjudicated reference standard, these findings concern reproducibility and agreement rather than diagnostic or screening performance and characterize a single LLM-based platform rather than generative AI in general.

## 1. Introduction

Morphological changes in the mandibular cortex are widely recognized as indicators of systemic bone metabolism abnormalities, including age-related changes and osteoporosis. In particular, the mandibular cortical index (MCI) has played an important role as a screening tool for bone quality assessment, as it can be readily evaluated on dental panoramic radiographs (DPR) [1,2]. The MCI is a classification system proposed by Klemetti et al., used to visually evaluate the continuity and degree of resorption of the cortical bone as a morphological indicator associated with osteoporosis risk [3]. Because osteoporosis is a silent chronic disease, diagnosis is often delayed, and the resulting low patient consultation rates have become a major public health concern [4,5]; there is growing interest in the potential of dental care, with MCI assessment playing a central role, to help address this problem. Nevertheless, MCI assessment is highly dependent on the observer’s experience, and inter-rater variability has been identified as a challenge [2,6].

In recent years, the application of artificial intelligence (AI) in medical imaging diagnostics has advanced rapidly, and its utility has been reported across various areas of dentistry, including caries diagnosis, detection of periodontal disease, implant treatment planning, and detection of jawbone lesions [7,8]. Two distinct lines of research should be distinguished in this context. The first comprises dedicated image-recognition models based on convolutional neural networks (CNNs) or vision transformers, which are trained end-to-end on radiographic data for a defined classification task. The second comprises general-purpose generative AI, in particular large language models (LLMs). Although LLMs are primarily designed for text processing, recent multimodal variants have begun to show capacity for image-based reasoning and are expected to serve as auxiliary tools for dental image interpretation [9], yet their performance in fine-grained morphological assessment remains an area of active investigation. Studies directly comparing generative AI with experienced dentists in evaluating subtle anatomical findings, such as those required for MCI classification, remain scarce [10]. Furthermore, although MCI classification is of considerable clinical significance in osteoporosis risk assessment and geriatric medicine, its accurate diagnosis requires a certain level of experience and image interpretation skill. Given the substantial inter-examiner and inter-facility variability in radiographic assessment, AI-based automated evaluation could enable consistent bone quality assessment and is expected to improve screening accuracy. It also has the potential to reduce the burden of complex image interpretation workflows and support the training of junior dentists.

Among currently available systems, however, institutional data-protection requirements precluded the use of open, externally hosted multimodal systems for patient radiographs. We therefore selected NotebookLM (Google), a closed-source generative AI tool designed primarily for document analysis and question answering. The aim of this study was to apply this tool to the task of MCI classification on DPR images and compare the results with those of eight dentists, in order to identify any tendencies in AI classification behaviour. We focused particularly on inter-rater and intra-rater reliability to examine whether AI possesses the reproducibility required for clinical application in MCI assessment. Accordingly, this study was designed as a descriptive reliability study rather than a formal equivalence trial. For descriptive purposes, we framed our expectation as the hypothesis that “the MCI classifications assigned by the generative AI tool and by dentists show no agreement beyond chance,” although this study was not designed to test this hypothesis formally through a prespecified primary comparison or inferential procedure. The findings of this study are expected to provide foundational data for the future implementation of AI as an auxiliary tool for dental image interpretation.

## 2. Materials and Methods

### 2.1. Study Design

This cross-sectional study evaluated the mandibular cortical index (MCI) classification of patients who visited the Nihon University School of Dentistry Mishima Dental Center. The study protocol was approved by the Ethics Committee of the Nihon University School of Dentistry (Approval No. EP25D026). The study was conducted in strict accordance with the Declaration of Helsinki (1975) [11], as revised in 2013, and followed the Strengthening the Reporting of Observational Studies in Epidemiology (STROBE) guidelines for observational studies [12]. As this study specifically concerns inter-rater and intra-rater reliability, we additionally followed relevant reporting elements of the Guidelines for Reporting Reliability and Agreement Studies (GRRAS) [13], including the reporting of the sampling frame, the number of eligible subjects, the selection procedure, rater blinding, and the specific agreement statistics used, as detailed in Section 2.2, Section 2.3 and Section 2.7.

### 2.2. Patient Selection and Data Sources

The subjects of this study were patients who visited the Nihon University School of Dentistry Mishima Dental Center between December 2015 and March 2022. Digital panoramic radiograph (DPR) images obtained from these patients were utilized for analysis. To ensure patient anonymity, the images were exported and saved as JPEG files, excluding DICOM metadata and personal annotations. The inclusion criteria were female patients aged 20 years or older who underwent DPR imaging as part of their initial diagnostic workup at the center. The exclusion criteria were as follows: (1) images taken at external facilities; (2) patients who were pregnant or breastfeeding; (3) images where morphological diagnosis of the mandibular cortex was not possible due to artifacts or suboptimal positioning; (4) a history of mandibular resection or reconstruction; (5) bone destruction due to neoplastic lesions; and (6) a history of radiotherapy to the head and neck region.

During the study period, 167 female patients aged 20 years or older met the inclusion criteria described above. From this pool, 100 images were randomly selected for inclusion in the study. Information on comorbidities, medication use, and osteoporosis status was available in the clinical records for some patients; however, these data were based on patient self-report or, for osteoporosis, on the presence or absence of a physician’s diagnostic note rather than a standardized diagnostic procedure such as DXA, and medication histories in particular were frequently incomplete or unreliable. As this study was designed to compare the reproducibility and agreement of MCI classification between human examiners and a generative AI tool, rather than to establish or validate an osteoporosis diagnosis, we did not systematically extract or report these variables; doing so would imply a level of clinical characterization that this observer-agreement study does not require and the underlying data quality does not support.

The primary outcome was to evaluate the inter-rater reliability of MCI classification between dentists and AI. The secondary outcome was to assess the inter-rater reliability among dentists with varying levels of clinical experience.

### 2.3. DPR Imaging Data

All DPR images utilized in this study were obtained as part of patients’ initial diagnostic workup at Mishima Dental Center, using the same radiographic unit (Veraviewepocs X700, Morita, Kyoto, Japan). A random sample of 100 images was selected and exported as JPEG files. Random selection was performed by assigning a random number generated in Microsoft Excel (RAND function) to each of the 167 eligible cases, sorting cases in ascending order of this random number, and selecting the 100 cases with the smallest random values. This sample size is consistent with that of previous studies on the reproducibility of the MCI [2,6]; no formal a priori sample-size or precision calculation was performed, as the study was designed as a descriptive reliability study, and the resulting precision of the kappa estimates should be judged from the confidence intervals reported alongside each coefficient. These images were individually embedded into a presentation software (Microsoft PowerPoint LTSC 2021, version 2408, Microsoft Corp., Redmond, WA, USA) to create a 100-page document.

**Figure 1:**
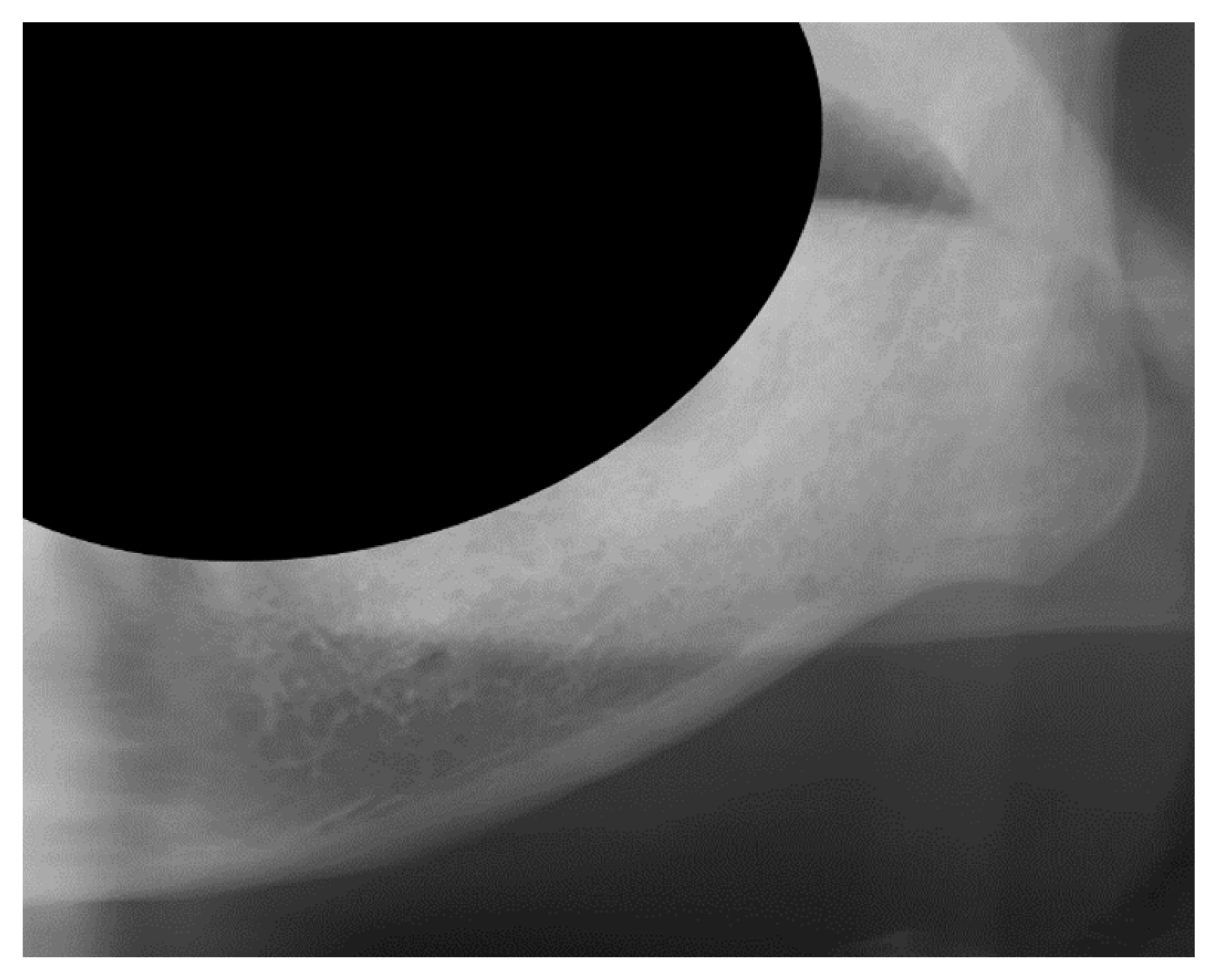
Cropped and masked panoramic radiograph prepared for MCI evaluation. To protect patient privacy, the image was trimmed at the midline to display only the left mandibular inferior cortex, and the dentition region was obscured with a black oval overlay to prevent age-related diagnostic bias. Patient identification data were removed, and images were stored as JPEG files without DICOM annotation.

**Figure 2:**
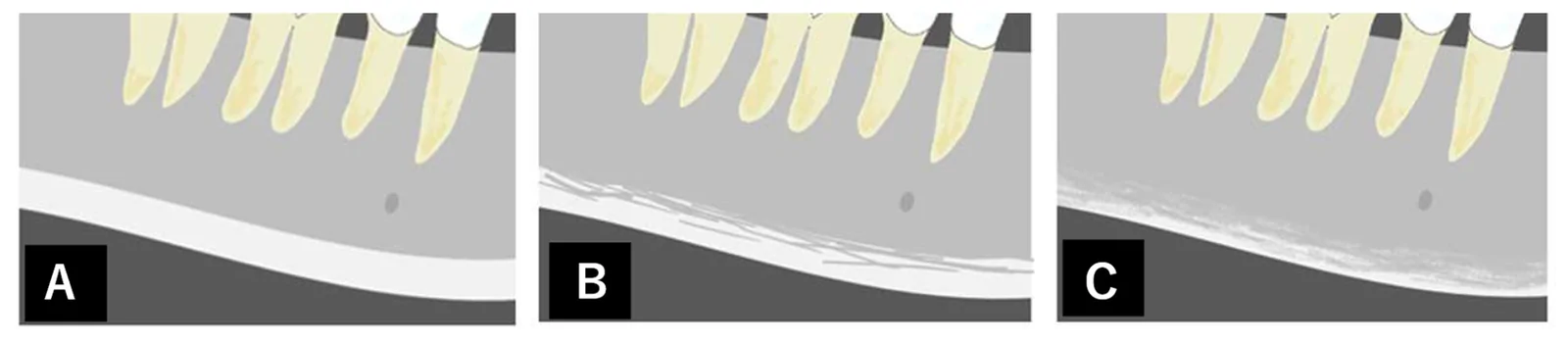
(**A**–**C**) Reference illustrations for MCI classification. (**A**) Class 1 (C1): smooth and intact endosteal margin; (**B**) Class 2 (C2): irregular endosteal margin with endosteal cortical resorption; (**C**) Class 3 (C3): heavy endosteal resorption with cortical layer discontinuity.

To ensure patient privacy and standardized evaluation, each DPR image was cropped to show only the inferior border of the left mandible. Specifically, the images were split at the midline, and the dental region was masked using a black elliptical overlay within the software (Figure 1). To minimize potential bias, as the number of remaining teeth might suggest a patient’s age, the same masking procedure was applied to all cases, including edentulous patients. A comparable masking of non-cortical regions of the panoramic radiograph has been applied in previous MCI evaluations [14]. Examiners, including the AI, were presented with the cropped and masked radiographic images only and were blinded to all clinical information, including patient age, medical history, medication use, and osteoporosis status; no information beyond the image itself and the reference illustrations for the MCI classification criteria was provided. Finally, reference illustrations for the MCI classification criteria were included on the first page, and the entire document was converted to a PDF format to serve as the standardized evaluation sample. The reference illustrations for the MCI classification criteria (Figure 2) were schematic drawings, adapted by the authors from previously published depictions of the Klemetti classification, rather than annotated radiographic examples; this approach was chosen to present the defining morphological features of each class clearly and without the potential ambiguity of case-specific radiographic variation, but we acknowledge that calibration using actual radiographic examples might have more closely approximated the images subsequently evaluated.

The complete set of modifications applied to the radiographs consisted of the following, and no others: (1) export from DICOM to JPEG with removal of all metadata and personal annotations, performed to anonymize the images; (2) splitting at the midline and cropping to the left inferior mandibular border, performed to remove identifying information and to standardize the field of assessment; (3) application of a black elliptical overlay over the dentition, performed to prevent the number of remaining teeth from biasing examiners toward a patient’s likely age; and (4) conversion of the assembled document to PDF, performed to provide an identical evaluation sample to all examiners and to the AI. No contrast enhancement, brightness normalization, sharpening, filtering, resampling, or any other deliberate image processing was applied to the cortical region at any stage; the only alterations to pixel values were those introduced by lossy compression during JPEG export and PDF conversion, as described below.

The exported JPEG images had a resolution of 2287 × 1024 pixels at 253 dpi with 8-bit greyscale depth, and were inserted into the presentation software without resizing. The image-quality setting of the presentation software was configured to “high fidelity,” so that no downsampling was applied at that stage. The assembled 100-page presentation (23.0 MB) was then converted to PDF (5.05 MB) using the standard export function; the compression parameters applied at JPEG export and during PDF conversion were not recorded. Examiners viewed the evaluation sample on their own computers; monitors were not calibrated, and although the PDF format permitted magnification of individual images, whether examiners made use of this facility was not recorded. These conditions reflect the circumstances under which panoramic radiographs are ordinarily reviewed in general dental practice, but they were not standardized across examiners.

### 2.4. Evaluation of the Mandibular Cortical Index (MCI)

Figure 2A–C presents the reference illustrations provided on the initial page of the evaluation sample. Based on the classification reported by Klemetti et al. [3], the morphology of the mandibular cortical bone at the inferior border was categorized into three classes:

- Class 1 (C1): The endosteal margin of the cortex is even and sharp on both sides.
- Class 2 (C2): The endosteal margin shows semilunar defects (lacunar resorption) or forms endosteal cortical residues.
- Class 3 (C3): The cortical layer is heavy with endosteal residues and is clearly porous.

To facilitate the evaluation process for the participants, these definitions were simplified as follows. Class 1 (C1) was described as having a smooth inner surface, Class 2 (C2) as having an irregular inner surface with linear resorption, and Class 3 (C3) as characterized by extensive resorption and porous changes throughout the cortical bone. It should be noted that the MCI is a qualitative classification of the morphology of the endosteal cortical margin and is distinct from the mandibular cortical width (MCW), which quantifies cortical thickness [2]; as a morphological index of the cortex distal to the mental foramen, the MCI can be reliably assessed on a single side, and all evaluations in this study were therefore standardized on the left inferior mandibular cortex.

### 2.5. Examiners: Dentists

The panel of examiners consisted of eight dentists with varying levels of clinical experience:

- A male periodontist (PER-M28; 28 years of clinical experience)
- A male endodontist (END-M33; 33 years)
- A female prosthodontist (PRO-F29; 29 years)
- A male prosthodontist (PRO-M19; 19 years)
- A male dental radiologist (RAD-REF; 14 years), who served as the reference examiner
- A male general practitioner (GP-M02; 2 years)
- A female postgraduate resident (PGR-F01; 1 year)
- A male postgraduate resident (PGR-M01; 1 year)

The clinical experience of each examiner is indicated within the respective identifiers (e.g., “28” in PER-M28 refers to 28 years of experience). The panel was intentionally composed of examiners with heterogeneous specialties and experience levels to reflect the realistic opportunistic-screening setting, in which the MCI is typically assessed by non-radiologist dentists rather than by radiologists. As no absolute gold standard exists for the diagnostic assessment of MCI, the results provided by the single dental radiologist (RAD-REF) were utilized as the reference data; the limitations of a single-reader reference are discussed in Section 4.3. We acknowledge that RAD-REF’s own classifications varied between sessions to a similar degree as those of the other examiners (see Section 3.2); this reflects the inherent difficulty of the classification task rather than a deficiency specific to this examiner, but it further underscores that RAD-REF’s judgements constitute a single, imperfect reference rather than a gold standard. The MCI classification was evaluated based on the reference illustrations provided on the first page of the sample file, and the results were recorded in separate Excel spreadsheets. Evaluations were conducted in two sessions: an initial session and a second session held at least two weeks later to minimize recall bias. Separate response sheets were used for each session, and the total time required for each evaluation was recorded.

### 2.6. Examiners: Generative AI

To evaluate the sample images, NotebookLM (Google LLC, Mountain View, CA, USA; accessed February 2026), a closed-source generative AI tool, was utilized to ensure the protection of personal information. As NotebookLM is a proprietary system, the specific underlying model version and Gemini variant employed could not be precisely identified at the time of the study. As the AI examiner, NotebookLM processed the sample PDF file following a specific protocol. After the file was uploaded, the following prompt was entered in Japanese to request the MCI assessment: “Please examine the images of the inferior border of the left mandible in this file and classify them according to the MCI criteria, referring to the reference illustrations on the first page. Please provide the results for each case as Class 1, Class 2, or Class 3 in text format.” The evaluation time for the AI was defined as the processing duration displayed within the browser environment. This measure captures only the computation performed by the system and excludes file upload, prompt entry, and transcription of the output; the evaluation times of the AI and of the dentists are therefore not directly comparable as workflow durations. As with the dental examiners, the AI evaluation was conducted in two separate sessions with an interval of at least two weeks to evaluate intra-examiner reliability. The prompt and the provided sample file were identical in both sessions. The prompt was fixed before the first session and was not modified thereafter; it was entered as a single instruction, without follow-up questions, iterative refinement, or few-shot examples beyond the reference illustrations contained on the first page of the sample file. Each session was conducted in a newly created notebook, so that no conversational context from the first session could be carried over to the second. No prompt-sensitivity analysis was undertaken, and the AI was run once per session rather than repeatedly within a session; the effect of alternative phrasings, and the run-to-run variability of the system under an identical prompt, therefore remain unknown.

NotebookLM was accessed using an institutional faculty account provided by the authors’ university, in accordance with the university’s internal guidance recommending the use of closed-source AI platforms, including NotebookLM, for tasks involving institutional or research-related data. No additional formal authorization beyond the institutional ethics committee approval (Section 2.1) was obtained specifically for this cloud-based tool; the university’s guidance was communicated as an administrative notice rather than a formal data-governance policy document. We acknowledge, as raised by the reviewer, that the use of a closed-source platform is not in itself synonymous with fully local or regulation-compliant data processing; our rationale rested on institutional guidance and the platform’s stated data-handling practices, rather than on independent verification of its backend infrastructure.

The exact timestamps of each session, the verbatim raw text output returned by the system for every case, and the specific NotebookLM account tier in effect at the time of analysis were not systematically archived beyond the classification results reported in Supplementary Tables S1 and S2. No cases produced missing or ambiguous output requiring special handling; all 100 cases received a Class 1, 2, or 3 classification in both sessions. Regarding data retention, we relied on the data-handling terms publicly disclosed by the developer for institutional NotebookLM accounts at the time of access; we did not independently verify server-side data retention practices beyond these published terms.

### 2.7. Statistical Analysis

**Figure 3:**
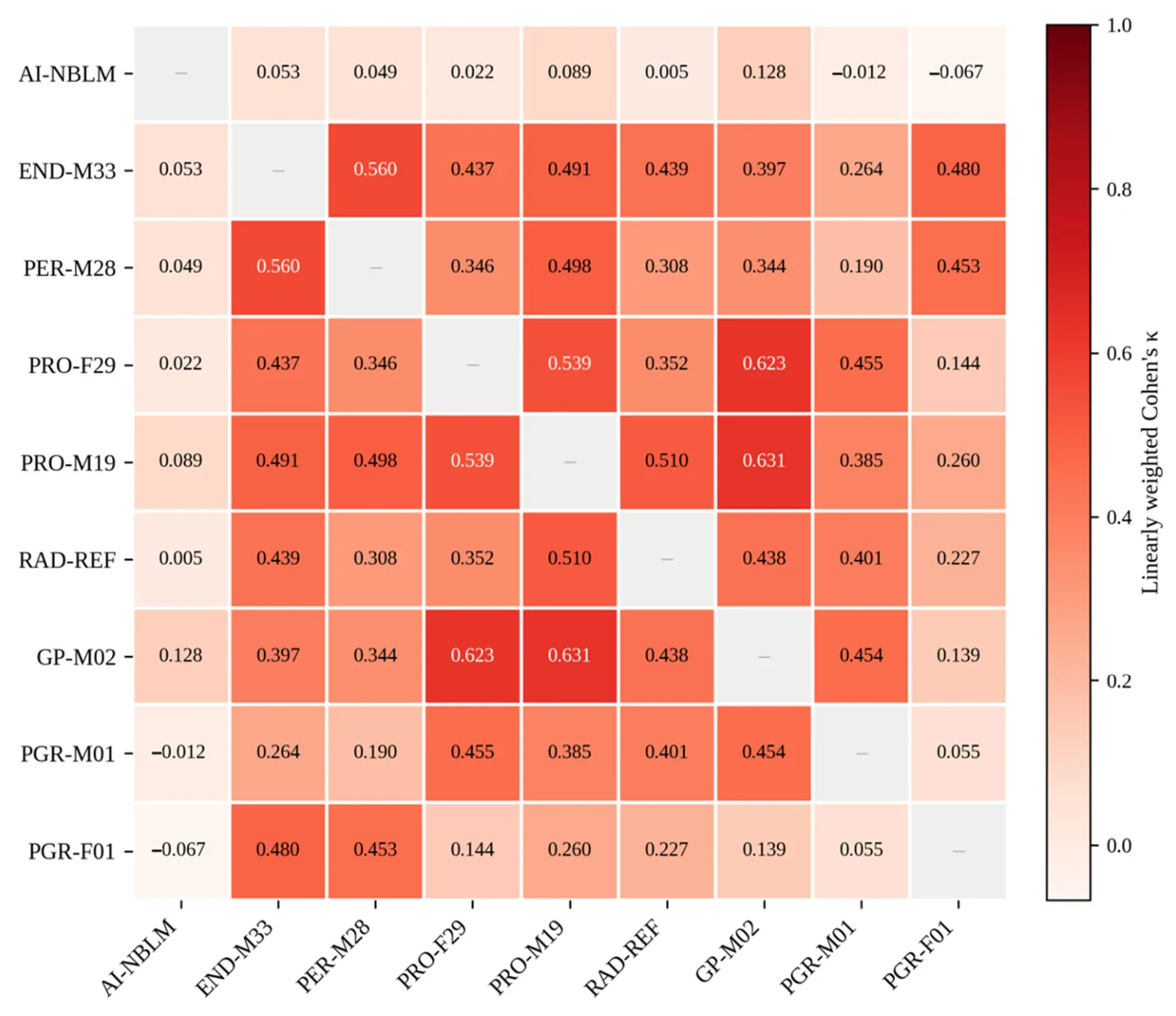
Heatmap of pairwise inter-examiner agreement (linearly weighted Cohen’s κ) from the first session. Darker red colors indicate higher agreement. The rows and columns corresponding to the generative AI (NotebookLM) show consistently low values, forming a pattern distinct from the dentist-only regions.

**Figure 4:**
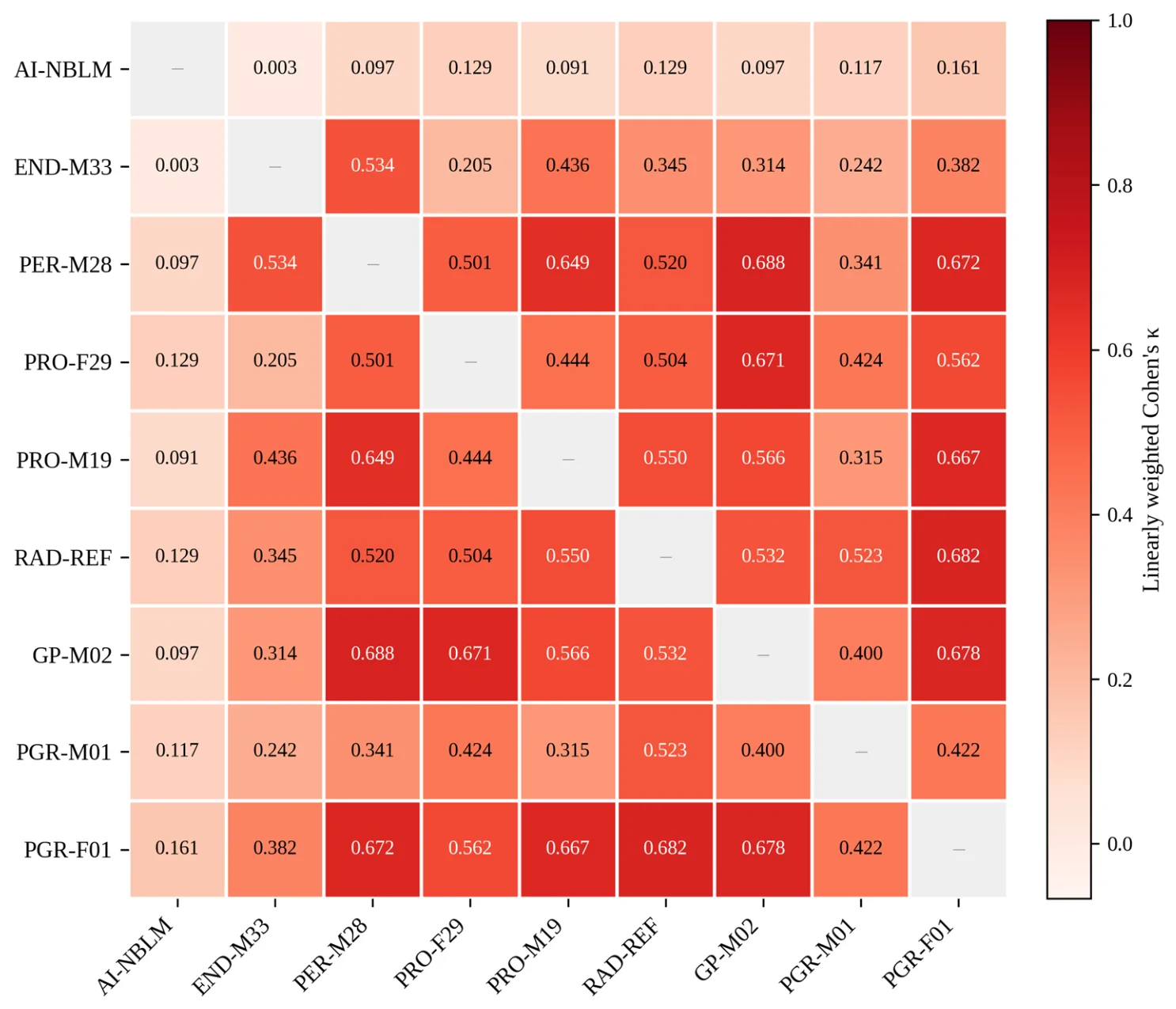
Heatmap of pairwise inter-examiner agreement (linearly weighted Cohen’s κ) from the second session. Compared with Figure 3, the high-agreement regions among dentists expanded, visually reflecting the convergence of diagnostic criteria through repeated evaluation. NotebookLM remained isolated in the low-agreement zone.

To evaluate the reliability of the MCI classification (Classes 1–3), the kappa coefficient was calculated. Since the MCI is an ordinal scale in which the degree of bone resorption increases as the class category increases, the analysis was performed using linear weights that reflect the distance of disagreement, rather than a simple kappa coefficient. To assess inter-rater reliability among all nine raters, including eight human raters and one generative AI (NotebookLM), the weighted Fleiss’ kappa coefficient was used. In addition, the weighted Cohen’s kappa coefficient was used to assess inter-rater reliability between the two raters and the agreement between two assessments by the same rater (intra-rater reliability) [15]. All analyses were performed using EZR (Saitama Medical Center, Jichi Medical University), a graphical user interface for R (The R Foundation for Statistical Computing, Vienna, Austria, version 4.0.0) [16], and 95% confidence intervals (95% CI) were calculated for each coefficient. Weighted Cohen’s κ and its 95% confidence interval were computed using the “Weighted kappa” function under the Statistical Analysis menu of EZR; the exact underlying R package and function name were not recorded at the time of analysis. No cases had missing classifications, so no missing-data procedure was required. The kappa coefficient is interpreted according to the Landis and Koch criteria: 0.00 or less is “Poor,” 0.01–0.20 is “Slight,” 0.21–0.40 is “Fair,” 0.41–0.60 is “Moderate,” 0.61–0.80 is “Substantial,” and 0.81–1.00 is “Almost perfect” [17]. These benchmarks are descriptive conventions for characterizing the extent of agreement and do not represent validated thresholds of clinical acceptability. In practical terms, “slight” agreement indicates that the concordance between two raters is little greater than would be expected by chance alone, whereas “substantial” agreement indicates that raters assign the same category in the large majority of cases and that discrepancies are usually confined to adjacent classes. For an ordinal index such as the MCI, disagreements between adjacent classes (for example, Class 1 versus Class 2) carry less clinical consequence than two-level discrepancies (Class 1 versus Class 3), since the latter reverse the interpretation from normal to severe cortical erosion; the linear weighting applied here reflects this distinction. No formal equivalence testing with a predefined equivalence margin was performed; accordingly, all comparisons in this study are descriptive assessments of agreement rather than tests of diagnostic equivalence or accuracy. Weighted Cohen’s κ can be affected by the marginal distribution of categories, a phenomenon known as the prevalence paradox [18]. To complement it, we additionally computed Gwet’s AC2 [19,20], a prevalence-adjusted agreement coefficient for ordinal data, together with the exact (unweighted) agreement rate. To support reproducibility, the kappa coefficients, confusion matrices, and Figure 3 and Figure 4 were independently recalculated using a Python 3.14 script provided in the Supplementary Materials (Code S1).

## 3. Results

### 3.1. Inter-Rater Reliability

**Figure 5:**
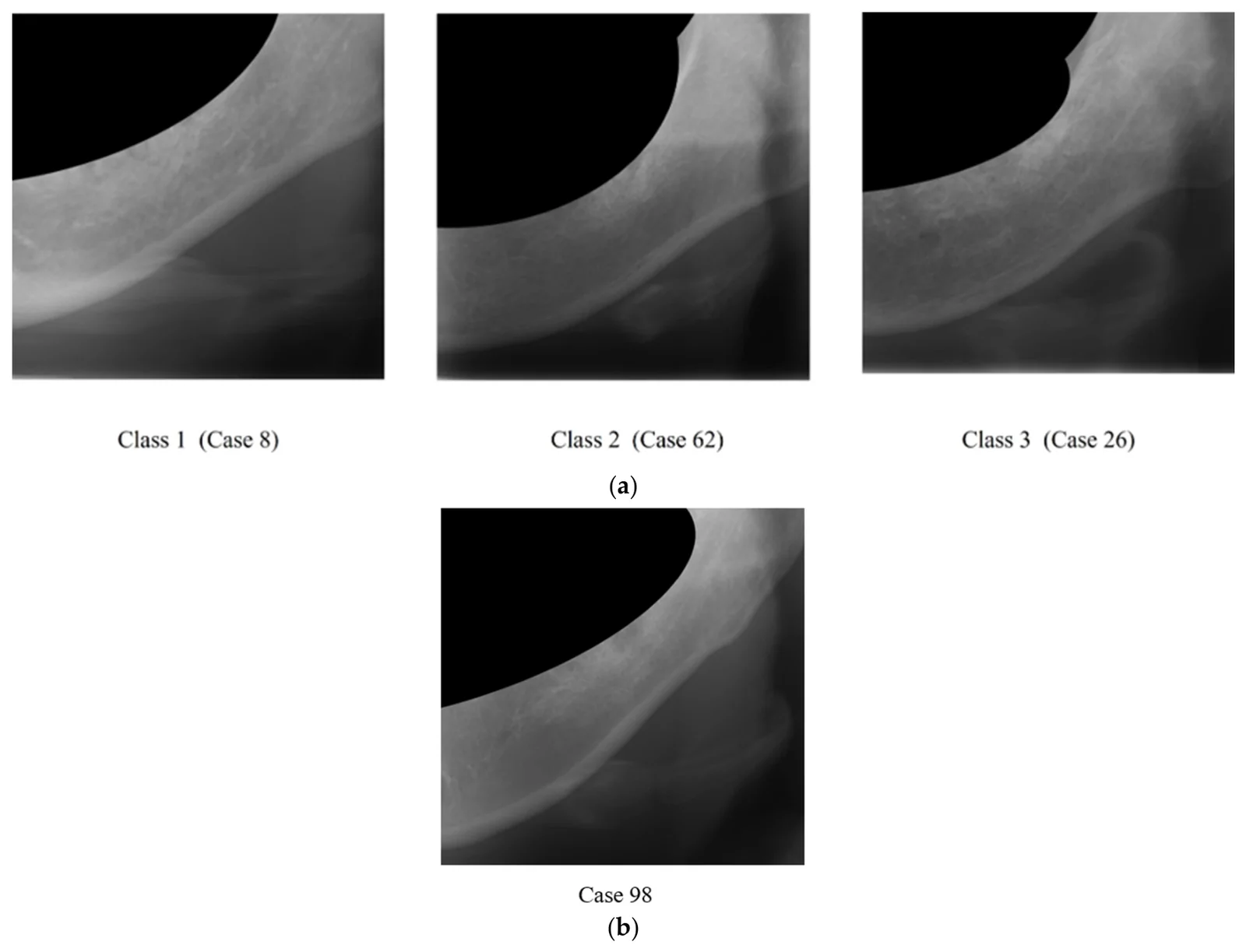
(**a**) Representative panoramic radiograph cases showing MCI Class 1, 2, and 3 on which all examiners reached consensus in at least one of the two evaluation sessions. (**b**) A representative case (Case 98) illustrating AI–human discordance. NotebookLM classified this case as Class 3 (severe) in both sessions, whereas the reference radiologist and the majority of the dentists classified it as Class 1 (normal). This two-level discrepancy, reproduced consistently across both evaluation sessions, illustrates the pattern of AI–dentist divergence characterized in Section 3.4 and Section 3.5.

Table 1 summarizes the results of the inter-examiner reliability analysis for MCI classification among the eight dentists and NotebookLM. The weighted Fleiss’ kappa coefficient for all examiners (eight dentists and NotebookLM) was 0.498 for the first session and 0.542 for the second session. According to the Landis and Koch criteria, these values indicated “moderate agreement.” In contrast, the inter-examiner agreement among the dentists alone was 0.606 for the first session and 0.627 for the second session, representing “substantial agreement.” When the AI was included in the analysis, the overall agreement rate tended to be slightly lower compared to the agreement among human examiners alone. Figure 5a illustrates representative cases of examiner consensus, while Figure 5b illustrates a representative case of AI–dentist discordance (Case 98).

### 3.2. Intra-Examiner Reliability and Evaluation Time

Table 2 summarizes the agreement between the two assessments by the same examiner and the time required for each session. The intra-examiner reliability for the NotebookLM was 0.987 [95% CI: 0.962–1.000], which was the highest among all examiners and represented “almost perfect” agreement. In contrast, the intra-examiner reliability among the dentists ranged from 0.257 to 0.761, showing substantial individual variation. The two dental residents were categorized into the “fair agreement” group, which was the lowest reliability level among all examiners. Even the dentist with the highest score achieved 0.761 (“substantial agreement”), indicating that the AI’s reproducibility exceeded that of every human examiner. No formal statistical comparison between kappa coefficients was performed. Notably, the reference radiologist (RAD-REF) also demonstrated substantial intra-examiner reliability (κ = 0.703), ranking second among all nine examiners; 19 of the 100 classifications differed between sessions, of which only one instance involved a two-level discrepancy (Class 1 versus Class 3), with the remainder confined to adjacent-class shifts.

The average time required to evaluate the 100 cases for the dentists ranged from a minimum of 5.5 min (RAD-REF) to a maximum of 20.5 min (PRO-F29). In comparison, the processing time for NotebookLM was 1.5 min on average, approximately six times shorter than the mean evaluation time of the dentists; these durations, however, were not measured under equivalent workflow conditions. Table 3 presents the distribution of MCI classifications (C1, C2, and C3) assigned by each examiner across both sessions. Notably, AI-NBLM showed a nearly identical class distribution between Session 1 (C1: 40%, C2: 42%, C3: 18%) and Session 2 (C1: 41%, C2: 41%, C3: 18%), further corroborating its exceptionally high intra-examiner reproducibility. In contrast, PGR-M01 showed a marked shift toward C1 classification in the second session (58% to 69%), suggesting a systematic classification bias.

### 3.3. Discrepancies in Diagnostic Criteria Between Dentists and Generative AI

Supplementary Table S3 presents the degree of agreement across the 36 possible pairings among examiners during the first session. The linearly weighted kappa coefficients among the dentists ranged from 0.055 to 0.631. Among these combinations, “substantial agreement” was observed between GP-M02 and PRO-M19 (0.631) and between GP-M02 and PRO-F29 (0.623), representing the highest reliability among the human pairings. Moreover, “moderate agreement” was the most frequent category, occurring in 13 pairs. Conversely, some pairings, such as the two dental residents (PGR-F01 and PGR-M01), showed only “slight agreement” (0.055), highlighting considerable individual variation in diagnostic judgement. In contrast, the agreement between NotebookLM and the dentists ranged from −0.067 to 0.128, indicating a very low level of consensus. The highest value for NotebookLM-dentist combinations was 0.128 (“slight agreement”) with GP-M02. However, the agreement values were negative for both dental residents (PGR-F01: −0.067; PGR-M01: −0.012), resulting in a “poor agreement” classification. These findings suggest that while certain combinations of dentists maintain a reliable level of consistency through shared expertise, the classifications produced by NotebookLM diverge substantially from those of the human experts for MCI classification. Notably, marked discrepancies were observed where the AI and human assessments were completely reversed, specifically between Class 1 (normal/mild) and Class 3 (severe). As kappa coefficients are sensitive to the marginal distribution of categories (the “prevalence paradox”) [18], and the category distributions differed between the AI and the dentists (Table 3), we additionally computed Gwet’s AC2 [19,20], a prevalence-adjusted agreement coefficient less susceptible to this effect, and the exact (unweighted) agreement rate. For the AI–dentist pairings in this session, Gwet’s AC2 ranged from 0.61 to 0.74 (mean 0.68), and exact agreement ranged from 24% to 48% (mean 39%), both considerably higher than the corresponding kappa values. This divergence indicates that a substantial proportion of the low kappa values reflects the skewed and differing category distributions between the AI and the dentists, rather than a complete absence of agreement; however, it does not alter the clinically important finding that Class 1–Class 3 (two-level) discrepancies, which reverse the interpretation from normal to severe, occurred in 10–18% of cases (Section 3.5).

### 3.4. Consistency and Persisting Discrepancies in the Second Evaluation Session

Supplementary Table S4 presents the agreement levels for the 36 possible examiner pairings during the second session. Among the pairings of dentists, the agreement levels ranged from “fair” (7 pairs, κ = 0.205–0.400) through “moderate” (14 pairs) to “substantial” (7 pairs, up to κ = 0.688). Particularly high agreement was observed between GP-M02 and PER-M28 (0.688) and between PGR-F01 and RAD-REF (0.682), suggesting that the dentists maintained highly consistent diagnostic criteria across sessions. Conversely, pairings involving PGR-M01 showed relatively low agreement (range: 0.242–0.523). This may be attributed to a specific bias in PGR-M01’s assessments; for instance, this examiner classified 69 out of 100 cases as “Class 1.” In contrast, the agreement between NotebookLM and human examiners remained low across all combinations (range: 0.003 [END-M33] to 0.161 [PGR-F01]). As in the first session, Gwet’s AC2 for the AI–dentist pairings (0.62 to 0.73, mean 0.70) and exact agreement (36% to 51%, mean 45%) were considerably higher than the corresponding kappa values, for the same reasons discussed in Section 3.3. Unlike the first session, no pairings were categorized as “poor”; however, all AI–human combinations remained within the “slight agreement” range. For all AI-related pairings, the lower limit of the 95% confidence interval was either zero or a negative value, indicating that NotebookLM’s agreement with the human examiners did not differ significantly from chance. Detailed analysis of these discrepancies revealed a recurring “two-level discrepancy” (where Class 1 [normal/mild] and Class 3 [severe] were interchanged) across all AI–dentist pairings. These results further demonstrate that the classifications produced by this tool diverge substantially from those of the human experts.

### 3.5. Agreement Between Individual Examiners and the Reference Standard

An analysis of the agreement between individual examiners and the dental radiologist (RAD-REF), who served as the reference standard, revealed several key trends. Among the group of dentists, the degree of agreement with the reference ranged from “fair” (weighted κ = 0.227; first session of PGR-F01) to “substantial” (weighted κ = 0.682; second session of PGR-F01) for most participants. In contrast, the agreement between NotebookLM and the reference was consistently low, with weighted kappa values of 0.005 in the first session and 0.129 in the second session, both of which were categorized as “poor.” These results suggest that while the AI demonstrates exceptionally high intra-examiner reproducibility, its classifications diverge substantially from the clinical diagnostic criteria utilized by dental professionals. Table 4 presents the confusion matrices for AI-NBLM versus RAD-REF across both sessions. In Session 1, agreement on the diagonal totaled 38 cases, while in Session 2, it totaled 45 cases, indicating consistently low concordance. Discordance between AI and RAD-REF was distributed relatively evenly across all three reference classes (61–67% of C1, C2, and C3 cases in Session 1; 54–64% in Session 2), rather than being concentrated in a single class. Notably, two-level discrepancies (C1↔C3 interchange) were observed in 13 cases in Session 1 and 11 cases in Session 2.

### 3.6. Qualitative Analysis of Diagnostic Disagreements

A two-level discrepancy, defined as a case where the AI classified an image as Class 3 (severe) while a dentist classified it as Class 1 (normal/mild) or vice versa, was observed across all dentist-AI pairings in both sessions. As shown in Table 5, the total number of two-level discrepancies ranged from 10 to 17 cases (10.0–17.0%) in Session 1 and from 11 to 18 cases (11.0–18.0%) in Session 2. These discrepancies were observed consistently across all AI–dentist pairings, regardless of the dentist’s experience level.

### 3.7. Specific Classification Bias and the Nature of AI Discrepancies

Among some postgraduate residents (PGRs), a systematic tendency, or classification bias, toward assigning cases to a specific category (e.g., Class 1) was observed. The discrepancies noted for NotebookLM, in contrast, did not follow such a consistent directional bias. Because the system is closed-source and no explainability or error-analysis method was applied, the cause of its discrepancies cannot be determined from the present data; possible explanations are considered in the Discussion.

### 3.8. Visualization of Inter-Examiner Agreement Using Heatmaps

The inter-examiner agreement (linearly weighted κ coefficients) for the first session was visualized using a heatmap (Figure 3). In this heatmap, the rows and columns corresponding to NotebookLM appeared in predominantly light tones, indicating consistently low agreement and forming a visual pattern distinct from the human examiners. Among the dentists, areas representing high agreement (indicated in the red spectrum) were concentrated among mid-career and senior practitioners, particularly involving PRO-M19, GP-M02, and PRO-F29. In contrast, the rows and columns associated with the postgraduate residents (PGR-M01 and PGR-F01) generally exhibited lighter colors. These visual gradients illustrate the pattern of agreement levels across examiner pairs, without implying that lower agreement reflects lower diagnostic competence.

Next, the inter-examiner agreement for the second session is presented as a heatmap in Figure 4. In this second heatmap, the red areas representing high agreement among the dentists were generally more extensive and intense compared to those in the first session. This visually confirms a convergence of diagnostic criteria among the human examiners as they gained more experience with the evaluation process. In contrast, the rows and columns corresponding to NotebookLM remained isolated as light-colored regions. This indicates that the AI’s divergence from the group of dentists was persistent and consistent across both evaluation sessions.

## 4. Discussion

In the present study, we evaluated the degree of agreement between a generative AI tool (NotebookLM) and eight dentists in the classification of the mandibular cortical index (MCI). The agreement between the AI and all human examiners remained at “slight agreement” or lower in both sessions, and the lower limit of the 95% confidence interval was zero or negative for all AI–dentist pairings. The null hypothesis, that the MCI classifications assigned by the AI tool and by dentists show no agreement beyond chance, could therefore not be rejected. Consequently, the MCI classifications produced by the NotebookLM implementation evaluated in this study cannot be regarded as reproducing those of dental professionals. As this study did not validate MCI classifications against DXA-derived bone mineral density or any other clinical gold standard, these findings concern reproducibility and inter-rater agreement, and do not permit any inference regarding diagnostic accuracy.

### 4.1. Comparison of Reproducibility and Inter-Rater Agreement

Before interpreting these findings, it is important to distinguish between the categories of artificial intelligence that are often grouped together under the term “AI” in dental imaging research. At least four categories should be considered: (i) large language model (LLM)-based systems, which are optimized for text and structured document processing and possess only limited or indirect visual reasoning capacity; (ii) multimodal vision-language models, which integrate a dedicated visual encoder with a language model and can perform general image reasoning; (iii) convolutional neural network (CNN)-based diagnostic algorithms, which are trained end-to-end for a defined image-classification task; and (iv) AI systems trained specifically on dental radiographs for a target diagnostic task, such as MCI classification or osteoporosis screening [21,22]. NotebookLM, the system evaluated in the present study, belongs to the first category. Accordingly, our findings characterize the behaviour of an LLM-based, document-oriented tool applied to a fine-grained morphological classification task, and they should not be extrapolated to categories (ii) to (iv), which employ fundamentally different architectures for visual recognition.

One possible explanation for the discrepancy between AI and human judgement is a limitation in NotebookLM’s image analysis capabilities, although this cannot be established from the present data. Because the underlying model version and multimodal processing pathway of NotebookLM were not disclosed by the developer and could not be identified (Section 2.6), the system is more accurately described as an opaque generative platform with an unspecified image-processing mechanism, rather than definitively characterized as possessing limited visual reasoning; if its processing pipeline resembles that of other LLM-based tools, which are primarily designed for text and structured document data, it may lack the specialized computer vision architecture required for the high-precision recognition of subtle morphological changes along the endosteal margin of the cortical bone [23,24]. The observed ‘two-level discrepancy,’ in which Class 1 and Class 3 classifications were interchanged, would be consistent with such a limitation in visual processing. This interpretation is necessarily hypothetical: because the system is proprietary and closed-source, and because no explainability analysis was performed, the actual basis of these discrepancies cannot be verified. Relatedly, we cannot rule out the possibility that the system’s classifications were influenced by non-visual cues such as page order or document structure within the uploaded file, rather than the radiographic content itself; no control condition (e.g., randomized page order or a text-only control file) was included to test this possibility, and we identify this as an important limitation and a direction for future methodological work. Conversely, NotebookLM demonstrated an intra-examiner reliability of 0.987, indicating that its reproducibility in response to an identical prompt is exceptionally high. This finding underscores the necessity of evaluating agreement with expert assessment and reproducibility as independent characteristics when assessing the clinical utility of LLM-based tools.

The nature of this reproducibility should not, however, be overstated. The AI returned an identical class for 99 of the 100 cases across the two sessions, and its high intra-examiner reliability is therefore better understood as the response stability of a largely deterministic system under an identical prompt than as reproducibility in the sense in which the term applies to human observers, who reconstruct a judgement on each occasion. Conducting each session in a newly created notebook excludes recall of the previous response as an explanation, but it does not establish that the system re-evaluated the images rather than converging on the same output for the same input. Response stability of this kind carries no implication of diagnostic competence.

The selection of a closed-source system, NotebookLM, for this study was primarily driven by ethical considerations regarding data security. While utilizing multimodal AI systems with advanced computer vision capabilities, such as GPT-4o or Gemini 1.5 Pro, might have been ideal from a purely technical perspective, these open-type systems pose a potential risk of data transmission to external servers. Consequently, obtaining approval from the institutional ethics committee was not feasible due to concerns over the protection of patient privacy. Compliance with Japan’s Act on the Protection of Personal Information, national medical information guidelines [25,26], and the EU’s General Data Protection Regulation (GDPR) [27] necessitates strict control over the external sharing of medical data. These instruments define general regulatory obligations and do not, in themselves, establish the compliance status of the specific NotebookLM account used in this study (Section 2.6). This remains a significant international challenge for the research and development of medical AI. Recently, there has been progress in on-premises AI systems and dedicated medical-grade closed systems that operate entirely within local infrastructures [28]. As technical solutions and legal frameworks evolve, we anticipate the establishment of environments where high-precision AI can be utilized in an ethically sound manner. Previous studies have reported that dedicated deep-learning-based diagnostic models demonstrate high accuracy in evaluating mandibular morphology, including MCI classification [21,22]. Therefore, comparative validation between such specialized systems and general-purpose generative AI remains a critical task for future research.

A further consideration concerns the external validity of our findings. Only a single commercially available platform was evaluated, and generative AI systems differ substantially from one another and are updated rapidly. Our results therefore cannot be generalized to GPT-based multimodal models, to Gemini Vision or Claude Vision, to dedicated dental AI software, or to future versions of NotebookLM itself, any of which may exhibit markedly different behaviour in fine-grained morphological classification. The present findings should accordingly be regarded as characterizing the version of NotebookLM accessed in February 2026, rather than the capabilities of generative AI in general.

### 4.2. Learning Effects in Human Examiners

Regarding diagnostic proficiency among the dentists, the average pairwise inter-examiner agreement (mean of linearly weighted Cohen’s κ from Table 4 and Table 5) improved from 0.386 to 0.491 between the first and second sessions, and the number of pairings demonstrating “substantial agreement” increased from two to seven. This convergence of diagnostic criteria, also visually confirmed by the heatmap, suggests a possible learning effect resulting from repeated evaluations, although the influence of memory from the first session cannot be entirely excluded. No structured training intervention was delivered between the sessions, and the same one hundred cases were presented in the same order in both sessions, without randomization; the improvement may therefore reflect familiarity with the task, recall of earlier judgements facilitated by the identical case order, or random variation, rather than a genuine gain in diagnostic skill. However, as the assessments were conducted only twice, longitudinal studies are required to identify the learning plateau and evaluate long-term diagnostic stability. Considerable individual variation was observed among the postgraduate residents; while PGR-F01 showed relatively higher inter-examiner agreement with experienced practitioners in the second session, this should be interpreted cautiously given the low intra-examiner reliability (κ = 0.257), which may reflect inconsistent application of diagnostic criteria rather than genuine learning. PGR-M01 showed relatively lower agreement with other examiners (Section 3.3), consistent with the classification bias noted in Section 3.7. Taken together, the present results have several implications for clinical practice. First, MCI assessment should continue to be performed by trained clinicians. The NotebookLM implementation evaluated here showed no agreement beyond chance with any human examiner, and its classifications were interchanged between Class 1 and Class 3 in 10–18% of cases across all AI–dentist pairings. A tool exhibiting such two-level discrepancies cannot be regarded as safe for preliminary screening, since a case classified as Class 3 by the reference examiner could be classified as Class 1 by the AI, or vice versa. Second, although a hybrid workflow combining AI-based initial screening with confirmatory diagnosis by a dentist is frequently proposed [29,30], our data do not support the deployment of an LLM-based tool in that role at present; such a workflow would require a system whose agreement with expert assessment is substantially higher than that observed here. Third, the added value of the system evaluated in this study lies not in classification but in reproducibility: its intra-examiner reliability (κ = 0.987) far exceeded that of every dentist, indicating that once a system attains adequate agreement with expert criteria, it could contribute to standardizing assessment across examiners and institutions. While AI could in principle serve as a training tool for residents by providing consistent feedback and standardized evaluation criteria [31,32], the NotebookLM implementation evaluated here does not meet the level of agreement with expert assessment required for such educational applications. From the perspective of standardizing assessment criteria across clinical facilities and promoting large-scale osteoporosis screening, the integration of computer-vision AI validated for this task remains highly significant [33,34].

### 4.3. Limitations

This study has several limitations. First, as no absolute gold standard exists for MCI classification, the results of a single dental radiologist (RAD-REF) were used as the reference standard. While this approach is consistent with previous studies in the field [2,6], the absence of an objective reference such as dual-energy X-ray absorptiometry (DXA)-based bone mineral density or a consensus diagnosis by multiple radiologists limits the interpretability of accuracy-related findings. In particular, a reference derived from an expert consensus panel or from independent duplicate readings by multiple oral radiologists would have reduced the subjectivity inherent in a single reader and is recommended for future studies. Second, the small sample size of 100 cases from a single facility, combined with the evaluation by only eight dentists, drawn from a single university department with only one or two examiners per specialty, may limit the generalizability of the findings and precludes statistically disentangling the effects of specialty from individual examiner variability. The study population comprised exclusively female patients aged 20 years or older, a choice justified by the higher prevalence of osteoporosis among postmenopausal women; whether comparable reproducibility would be observed in mixed-sex populations or in younger individuals, in whom cortical morphology is generally less advanced, remains to be determined. Furthermore, all radiographs were acquired with a single imaging unit under a uniform protocol, and differences in radiographic systems, image quality, exposure parameters, and patient populations may influence both human and AI reproducibility. Third, due to ethical constraints, a direct comparison with the latest AI systems specialized in computer vision was not feasible; therefore, these findings are restricted to a specific general-purpose LLM (NotebookLM) as accessed in February 2026, and cannot be generalized to other generative AI platforms or to subsequent versions of the same system. Additionally, the case order was identical in both sessions rather than randomized, which may have facilitated recall-based rather than genuine learning-based improvement; a detailed analysis of the specific causes of AI classification discrepancies (such as identifying focal points) and a longitudinal assessment of the stability of the observed learning effects among dentists remain insufficient. Fourth, only the left inferior mandibular cortex was evaluated; although the MCI is a morphological index that can be assessed unilaterally, this approach cannot capture potential left–right asymmetry in cortical morphology, and bilateral evaluation may be considered in future studies. Fifth, although no downsampling was applied within the presentation software and no operation altering greyscale values was performed deliberately, the images were subject to lossy JPEG compression at export and to further compression during PDF conversion, and these parameters were not recorded; a loss of fine cortical detail therefore cannot be excluded. Viewing conditions were likewise not standardized, as examiners used their own uncalibrated monitors. Sixth, the verbatim raw outputs returned by the system and the exact execution timestamps of each session were not archived, and the precise version of the underlying proprietary model could not be identified (Section 2.6). Exact computational reproducibility of the AI classifications reported here is therefore not achievable, and these results should be regarded as a record of the behaviour observed under the conditions described rather than as a computationally reproducible measurement. Future research involving large-scale validation across multiple institutions and a wider variety of AI systems is warranted to address these challenges.

## 5. Conclusions

This study compared the reproducibility of MCI classification on dental panoramic radiographs between a single, closed-source generative AI platform (NotebookLM) and eight dentists of varying clinical experience, using a fixed prompt and workflow. The AI demonstrated markedly higher intra-examiner reproducibility (κ = 0.987) than any human examiner. However, its classifications showed no agreement beyond chance with those of the dentists, and Class 1 and Class 3 were interchanged in 10–18% of cases. The NotebookLM implementation available during the study, under the conditions evaluated here, produced stable but discordant outputs relative to the dentists and cannot be regarded as suitable for MCI classification.

These findings should be interpreted as characterizing an LLM-based, document-oriented tool rather than generative AI as a whole, and they cannot be generalized to multimodal vision-language models, CNN-based diagnostic algorithms, or systems trained specifically on dental radiographs. High reproducibility and agreement with expert assessment are independent properties, and the former does not compensate for the absence of the latter. Comparative validation against computer-vision systems developed and validated for this task, conducted within an ethically compliant environment, remains an essential objective for future research.

## Figures and Tables

**Table 1:** Overall inter-examiner reliability (weighted Fleiss’ κ) for MCI classification. Agreement levels across all nine examiners (eight dentists and NotebookLM) and for the dentists only are shown for both sessions, including 95% confidence intervals.

	1st Session	2nd Session
All Examiners (AI + Dentists)	0.498	0.542
	[0.441, 0.555]	[0.485, 0.598]
Dentists Only	0.606	0.627
	[0.548, 0.664]	[0.571, 0.683]

**Table 2:** Intra-examiner reliability (weighted Cohen’s κ) and mean evaluation time per examiner. Results are based on 100 panoramic radiographs across two evaluation sessions.

Examiner ID	Weighted Cohen’s κ [95% CI]	Interpretation	Time 1 (min)	Time 2 (min)	Mean Time (min)
AI-NBLM	0.987 [0.962, 1.000]	Almost perfect	2	1	1.5
END-M33	0.685 [0.578, 0.792]	Substantial	5	7	6
PER-M28	0.64 [0.521, 0.758]	Substantial	7	6	6.5
PRO-F29	0.645 [0.512, 0.778]	Substantial	30	11	20.5
RAD-REF	0.703 [0.589, 0.817]	Substantial	6	5	5.5
PRO-M19	0.761 [0.651, 0.871]	Substantial	10	9	9.5
GP-M02	0.619 [0.466, 0.772]	Substantial	8	6	7
PGR-M01	0.416 [0.252, 0.581]	Fair	15	15	15
PGR-F01	0.257 [0.108, 0.406]	Fair	6	8	7

**Table 3:** MCI class distribution (C1, C2, and C3) by examiner for Session 1 and Session 2. Values are presented as number of cases (n) and percentage (%).

Session 1							Session 2						
Examiner	C1 (n)	C1 (%)	C2 (n)	C2 (%)	C3 (n)	C3 (%)	Examiner	C1 (n)	C1 (%)	C2 (n)	C2 (%)	C3 (n)	C3 (%)
AI-NBLM	40	40	42	42	18	18	AI-NBLM	41	41	41	41	18	18
RAD-REF	58	58	33	33	9	9	RAD-REF	54	54	35	35	11	11
END-M33	28	28	46	46	26	26	END-M33	26	26	45	45	29	29
PER-M28	23	23	45	45	32	32	PER-M28	28	28	51	51	21	21
PRO-F29	36	36	54	54	10	10	PRO-F29	40	40	54	54	6	6
PRO-M19	34	34	54	54	12	12	PRO-M19	32	32	54	54	14	14
GP-M02	43	43	50	50	7	7	GP-M02	32	32	55	55	13	13
PGR-M01	58	58	35	35	7	7	PGR-M01	69	69	24	24	7	7
PGR-F01	15	15	47	47	38	38	PGR-F01	45	45	37	37	18	18

**Table 4:** Confusion matrices of MCI classifications by AI-NBLM versus RAD-REF for Session 1 and Session 2. Diagonal cells indicate agreement; off-diagonal cells indicate disagreement. Two-level discrepancies correspond to the C1↔C3 interchange.

AI-NBLM vs. RAD-REF (Session 1)				
AI-NBLM/RAD-REF	C1	C2	C3	Row Total
C1	22	16	2	40
C2	25	13	4	42
C3	11	4	3	18
Col Total	58	33	9	100
**AI-NBLM vs. RAD-REF (Session 2)**				
C1	25	12	4	41
C2	22	16	3	41
C3	7	7	4	18
Col Total	54	35	11	100

**Table 5:** Two-level discrepancies (C1↔C3 interchange) between AI-NBLM and each dentist examiner for Session 1 and Session 2. Values indicate the number of cases in which AI-NBLM classified an image as C1 while the dentist classified it as C3 (C1 to C3), or vice versa (C3 to C1), along with the total count and percentage.

Session 1				
Dentist	C1 (AI)→C3 (Dentist)	C3 (AI)→C1 (Dentist)	Total (n)	Total (%)
RAD-REF	2	11	13	13
END-M33	11	2	13	13
PER-M28	15	2	17	17
PRO-F29	6	6	12	12
PRO-M19	5	5	10	10
GP-M02	4	6	10	10
PGR-M01	4	10	14	14
PGR-F01	15	0	15	15
**Session 2**				
RAD-REF	4	7	11	11
END-M33	15	3	18	18
PER-M28	9	4	13	13
PRO-F29	4	8	12	12
PRO-M19	8	4	12	12
GP-M02	5	6	11	11
PGR-M01	2	10	12	12
PGR-F01	7	8	15	15

## Data Availability

The original contributions presented in this study are included in the article/Supplementary Material. Further inquiries can be directed to the corresponding author.

## Supplementary Materials

The following supporting information can be downloaded at https://www.mdpi.com/article/10.3390/clinpract16080142/s1. Table S1: Raw MCI classification data (Session 1); Table S2: Raw MCI classification data (Session 2); Table S3: Pairwise inter-examiner agreement matrix, linearly weighted Cohen’s κ (Session 1); Table S4: Pairwise inter-examiner agreement matrix, linearly weighted Cohen’s κ (Session 2); Code S1: Python script for independent verification of kappa coefficients, confusion matrices, and Figure 3 and Figure 4.
