# Supplementary material for: Reproducibility of Mandibular Cortical Index Classification Among Dental Examiners and a Single Generative AI Platform: An Observer Agreement Study
Source: Clin Pract. 2026 Jul 31;16(8):142. doi: 10.3390/clinpract16080142 (PMC13511330; doi:10.3390/clinpract16080142)
Supplement: Supplementary file 1 [file clinpract-16-00142-s001.zip › Supplementary_Code_S1_Kappa_and_Figure_Verification.pdf]

Supplementary Code S1: Kappa and Figure Verification Script

```python
"""
Supplementary Code S1: Verification of Kappa Coefficients and Figures
Reproducibility of Mandibular Cortical Index Classification Among Dental
Examiners and a Generative AI Tool: An Observer Agreement Study

This script reads the raw classification data (Supplementary Tables S1 and
S2) and independently recomputes the weighted Cohen's kappa coefficients,
confusion matrices, and two-level discrepancy counts reported in the
manuscript (Table 2, Table 6, Table 7, and Supplementary Tables S3-S4). It
also regenerates Figures 4 and 5.

All coefficients reported in the manuscript, including the overall
weighted Fleiss' kappa values in Table 1, were originally calculated and
verified using EZR (Saitama Medical Center, Jichi Medical University), a
graphical user interface for R (version 4.0.0). This script provides an
independent, machine-readable verification for the weighted Cohen's kappa
statistics, which are reproduced here exactly.

LIMITATION: The overall weighted Fleiss' kappa values reported in Table 1
(all examiners and dentists-only) are NOT re-derived in this script. These
values were computed and verified in EZR using its built-in weighted
agreement procedure; several published generalizations of weighted Fleiss'
kappa exist (e.g., Conger 1980; Fleiss, Cohen & Everitt 1969), and we were
unable to confirm which exact formulation matches EZR's implementation
without access to its internal source. Rather than present an unverified
formula, we report this limitation transparently. Table 1 values should be
treated as verified through EZR, as stated in Section 2.7 of the
manuscript.

Requirements: pandas, numpy, scikit-learn, matplotlib
    pip install pandas numpy scikit-learn matplotlib

Usage:
    python Supplementary_Code_S1_Kappa_and_Figure_Verification.py \
        --session1 Supplementary_Table_S1_Raw_MCI_Classification_Session1.xlsx \
        --session2 Supplementary_Table_S2_Raw_MCI_Classification_Session2.xlsx
"""

import argparse
import itertools
import numpy as np
import pandas as pd
from sklearn.metrics import cohen_kappa_score, confusion_matrix

EXAMINER_ORDER = [
    "AI-NBLM", "END-M33", "PER-M28", "PRO-F29",
    "RAD-REF", "PRO-M19", "GP-M02", "PGR-M01", "PGR-F01",
]
LABELS = [1, 2, 3]


def load_session(path):
    """Load a raw classification file (xlsx or csv) and standardize columns."""
    if str(path).lower().endswith(".csv"):
        df = pd.read_csv(path)
    else:
        df = pd.read_excel(path)
    df.columns = [str(c).strip() for c in df.columns]
```

```python
    df = df[["Case"] + EXAMINER_ORDER].copy()
    for col in EXAMINER_ORDER:
        df[col] = df[col].astype(int)
    return df.sort_values("Case").reset_index(drop=True)


def weighted_kappa(a, b, labels=LABELS):
    """Linearly weighted Cohen's kappa point estimate (matches manuscript exactly)."""
    return cohen_kappa_score(a, b, weights="linear", labels=labels)


def build_table2(s1, s2):
    """Intra-examiner reliability (weighted Cohen's kappa between Session 1 and 2)."""
    rows = []
    for examiner in EXAMINER_ORDER:
        k = weighted_kappa(s1[examiner], s2[examiner])
        n_changed = int((s1[examiner].values != s2[examiner].values).sum())
        rows.append({
            "Examiner": examiner,
            "Weighted kappa (Session1 vs Session2)": round(k, 3),
            "Classifications changed / 100": n_changed,
        })
    return pd.DataFrame(rows)


def build_pairwise_matrix(df):
    """All 36 pairwise weighted Cohen's kappa values within one session."""
    rows = []
    for a, b in itertools.combinations(EXAMINER_ORDER, 2):
        k = weighted_kappa(df[a], df[b])
        rows.append({"Examiner A": a, "Examiner B": b, "kappa": round(k, 3)})
    return pd.DataFrame(rows)


def build_confusion(df, rater_a="AI-NBLM", rater_b="RAD-REF"):
    cm = confusion_matrix(df[rater_a], df[rater_b], labels=LABELS)
    return pd.DataFrame(cm, index=[f"{rater_a}=C{c}" for c in LABELS],
                        columns=[f"{rater_b}=C{c}" for c in LABELS])


def two_level_discrepancies(df, rater_a="AI-NBLM", rater_b="RAD-REF"):
    a, b = df[rater_a], df[rater_b]
    return int((((a == 1) & (b == 3)) | ((a == 3) & (b == 1))).sum())


def make_heatmap(df, title, outfile, vmin, vmax):
    import matplotlib
    matplotlib.use("Agg")
    import matplotlib.pyplot as plt
    try:
        plt.rcParams["font.family"] = "Liberation Serif"
    except Exception:
        pass

    n = len(EXAMINER_ORDER)
    mat = np.full((n, n), np.nan)
    for i, a in enumerate(EXAMINER_ORDER):
        for j, b in enumerate(EXAMINER_ORDER):
            if i == j:
                continue
            if i < j:
                k = weighted_kappa(df[a], df[b])
                mat[i, j] = k
```

```python
                mat[j, i] = k

    fig, ax = plt.subplots(figsize=(7.2, 6.4))
    masked = np.ma.masked_invalid(mat)
    cmap = plt.cm.Reds
    cmap.set_bad(color="#eeeeee")
    im = ax.imshow(masked, vmin=vmin, vmax=vmax, cmap=cmap)
    ax.set_xticks(range(n)); ax.set_xticklabels(EXAMINER_ORDER, rotation=45, ha="right", fontsize=10)
    ax.set_yticks(range(n)); ax.set_yticklabels(EXAMINER_ORDER, fontsize=10)
    for i in range(n):
        for j in range(n):
            v = mat[i, j]
            if np.isnan(v):
                ax.text(j, i, "-", ha="center", va="center", fontsize=10, color="#999999")
            else:
                norm = (v - vmin) / (vmax - vmin)
                color = "white" if norm > 0.55 else "black"
                ax.text(j, i, f"{v:.3f}", ha="center", va="center", fontsize=8.5, color=color)
    cbar = fig.colorbar(im, ax=ax, fraction=0.046, pad=0.04)
    cbar.set_label("Linearly weighted Cohen's kappa", fontsize=10)
    ax.set_title(title, fontsize=12)
    for spine in ax.spines.values():
        spine.set_visible(False)
    ax.set_xticks(np.arange(-0.5, n, 1), minor=True)
    ax.set_yticks(np.arange(-0.5, n, 1), minor=True)
    ax.grid(which="minor", color="white", linewidth=1.5)
    ax.tick_params(which="minor", bottom=False, left=False)
    plt.tight_layout()
    plt.savefig(outfile, dpi=300, facecolor="white")
    plt.close()


def main():
    parser = argparse.ArgumentParser(description="Verify weighted kappa statistics and regenerate figures.")
    parser.add_argument("--session1", required=True, help="Path to Supplementary Table S1 (Session 1 raw data)")
    parser.add_argument("--session2", required=True, help="Path to Supplementary Table S2 (Session 2 raw data)")
    parser.add_argument("--outdir", default=".", help="Output directory for generated tables/figures")
    args = parser.parse_args()

    s1 = load_session(args.session1)
    s2 = load_session(args.session2)

    print("NOTE: Table 1 (overall weighted Fleiss' kappa) is not reproduced by this script.")
    print("      Table 1 values were calculated and verified using EZR (see Section 2.7 of the manuscript).\n")

    print("=== Table 2: Intra-examiner reliability (weighted Cohen's kappa) ===")
    t2 = build_table2(s1, s2)
    print(t2.to_string(index=False))
    t2.to_csv(f"{args.outdir}/verification_Table2.csv", index=False)

    print("\n=== Supplementary Table S3: Pairwise kappa matrix, Session 1 (36 pairs) ===")
    s3 = build_pairwise_matrix(s1)
    s3.to_csv(f"{args.outdir}/verification_Table_S3.csv", index=False)
    print(s3.to_string(index=False))

    print("\n=== Supplementary Table S4: Pairwise kappa matrix, Session 2 (36 pairs) ===")
    s4 = build_pairwise_matrix(s2)
    s4.to_csv(f"{args.outdir}/verification_Table_S4.csv", index=False)
    print(s4.to_string(index=False))

    print("\n=== Table 6: Confusion matrix, AI-NBLM vs RAD-REF ===")
    for name, df in [("Session1", s1), ("Session2", s2)]:
        cm = build_confusion(df)
```

```python
        print(f"\n{name}:")
        print(cm.to_string())
        cm.to_csv(f"{args.outdir}/verification_Table6_{name}.csv")

    print("\n=== Table 7: Two-level discrepancies (Class 1 <-> Class 3), AI-NBLM vs RAD-REF ===")
    for name, df in [("Session 1", s1), ("Session 2", s2)]:
        n_two_level = two_level_discrepancies(df)
        print(f"{name}: {n_two_level} cases")

    print("\n=== Generating Figures 4 and 5 ===")
    all_vals = pd.concat([s3["kappa"], s4["kappa"]])
    vmin, vmax = float(all_vals.min()), 1.0
    make_heatmap(s1, "Figure 4. Pairwise inter-examiner agreement (weighted Cohen's kappa), Session 1",
                 f"{args.outdir}/verification_Figure4.png", vmin, vmax)
    make_heatmap(s2, "Figure 5. Pairwise inter-examiner agreement (weighted Cohen's kappa), Session 2",
                 f"{args.outdir}/verification_Figure5.png", vmin, vmax)
    print(f"Figures saved to {args.outdir}/verification_Figure4.png and verification_Figure5.png")

    print("\nAll values above (Table 2, Tables S3-S4, Table 6, Table 7, Figures 4-5) should match")
    print("the manuscript exactly. Table 1 is verified separately via EZR (see note above).")


if __name__ == "__main__":
    main()
```
