# Supplementary material for: Reproducibility of Mandibular Cortical Index Classification Among Dental Examiners and a Single Generative AI Platform: An Observer Agreement Study
Source: Clin Pract. 2026 Jul 31;16(8):142. doi: 10.3390/clinpract16080142 (PMC13511330; doi:10.3390/clinpract16080142)
Supplement: Supplementary file 1 [file clinpract-16-00142-s001.zip › Supplementary_Table_S1_Raw_MCI_Classification_Session1.pdf]

Case	AI-NBLM	END-M33	PER-M28	PRO-F29	RAD-REF	PRO-M19	GP-M02	PGR-M01	PGR-F01
1	1	2	2	2	1	2	2	2	2
2	1	1	1	1	1	1	1	1	1
3	2	1	2	2	1	2	2	2	1
4	2	2	3	2	2	2	2	2	2
5	2	3	3	2	3	3	2	2	3
6	1	1	2	1	1	1	1	1	1
7	1	2	2	1	1	1	1	1	2
8	1	1	1	1	1	1	1	1	1
9	2	2	2	2	1	2	2	2	1
10	3	2	3	2	2	2	2	1	2
11	2	3	3	2	2	2	2	2	3
12	1	1	3	1	1	1	1	1	2
13	1	3	3	3	2	2	2	3	3
14	1	2	1	1	1	1	2	2	2
15	2	1	1	1	1	1	1	1	1
16	2	2	2	1	1	1	1	1	3
17	1	1	2	1	1	1	1	1	2
18	1	1	1	2	1	2	1	1	2
19	2	1	1	1	1	1	1	1	3
20	1	2	2	2	1	1	1	1	3
21	3	3	2	1	1	1	1	1	3
22	2	3	2	2	1	2	2	2	2
23	3	2	2	2	1	1	2	2	2
24	1	3	3	2	2	2	2	2	3
25	2	3	3	3	2	2	2	2	3
26	3	3	3	3	3	3	3	3	3
27	2	3	2	2	3	2	3	2	3
28	3	3	3	2	2	2	2	2	3
29	2	3	3	2	2	2	2	1	3
30	1	1	2	1	1	1	1	1	2
31	1	1	1	1	1	1	1	1	2
32	1	2	1	1	1	1	1	1	2
33	2	1	1	1	1	1	1	1	2
34	2	1	2	2	1	2	2	2	1
35	1	2	2	3	1	2	2	3	3
36	1	3	3	3	3	2	2	2	3
37	2	2	2	2	2	2	2	2	3
38	3	3	3	2	1	3	2	1	3
39	1	3	3	2	2	2	2	2	3
40	1	2	2	2	2	2	1	2	2
41	1	1	2	1	1	1	1	1	2
42	1	1	1	1	1	1	1	1	2
43	3	2	3	2	1	2	2	2	2
44	3	3	3	3	3	3	3	2	3
45	2	3	2	3	2	2	2	2	3
46	2	2	1	2	1	2	2	1	1
47	1	3	3	2	1	3	2	1	3
48	1	2	2	1	2	2	1	2	2
49	2	2	2	1	1	1	1	1	3
50	1	2	3	2	2	2	2	2	3
51	1	1	1	1	1	1	1	1	2
52	1	3	2	2	2	2	1	2	2
53	1	2	1	1	2	1	1	1	2
54	1	2	2	2	1	2	1	1	2
55	2	3	3	1	3	3	2	1	3
56	2	2	2	2	2	1	1	1	2
57	2	2	2	2	2	2	2	1	2
58	2	1	2	1	1	1	1	1	2
59	1	2	2	2	1	2	2	1	2
60	2	2	1	2	1	2	1	2	1
61	1	2	3	2	2	2	2	2	2
62	2	2	2	2	2	2	2	2	2
63	2	2	1	2	1	1	1	1	1
64	2	2	2	2	1	2	2	1	2
65	3	2	2	1	1	1	1	1	2
66	1	3	3	2	3	3	2	3	3
67	2	1	1	2	1	1	1	1	1
68	2	3	3	1	3	3	2	1	3
69	2	2	2	2	2	2	2	2	1
70	3	1	2	1	1	2	1	1	2
71	1	2	3	2	2	3	3	1	3
72	2	2	2	1	1	1	1	1	3
73	2	2	2	1	2	2	2	1	3
74	2	1	1	1	1	1	1	1	2
75	1	2	2	2	2	2	1	1	3
76	2	1	2	2	1	2	2	1	1
77	2	2	2	2	1	2	2	2	2
78	2	2	3	2	2	2	2	1	2
79	2	2	3	2	1	2	2	1	3
80	3	2	3	2	1	2	2	1	2
81	1	2	3	3	2	2	2	2	3
82	2	1	1	1	1	1	1	1	1
83	2	1	2	1	1	2	1	1	3
84	2	1	1	2	1	1	2	1	2
85	1	3	3	3	2	2	3	3	2
86	3	2	1	1	1	1	1	1	2
87	1	3	3	3	2	3	3	2	3
88	2	1	1	1	1	2	1	3	2
89	1	3	3	2	2	3	3	1	3
90	3	2	2	1	1	2	1	1	2
91	3	2	2	2	2	2	2	1	2
92	3	2	2	2	2	2	2	2	2
93	3	3	3	2	3	3	2	2	3
94	2	1	1	1	1	1	1	1	1
95	2	2	2	2	2	2	2	3	2
96	1	1	2	2	1	2	2	1	2
97	1	2	2	2	1	2	1	1	2
98	3	1	1	1	1	1	1	1	3
99	3	2	2	2	1	2	2	2	2
100	1	3	3	2	2	2	2	2	3
