# Supplementary material for: Reproducibility of Mandibular Cortical Index Classification Among Dental Examiners and a Single Generative AI Platform: An Observer Agreement Study
Source: Clin Pract. 2026 Jul 31;16(8):142. doi: 10.3390/clinpract16080142 (PMC13511330; doi:10.3390/clinpract16080142)
Supplement: Supplementary file 1 [file clinpract-16-00142-s001.zip › Supplementary_Table_S2_Raw_MCI_Classification_Session2.pdf]

Case	AI-NBLM	END-M33	PER-M28	PRO-F29	RAD-REF	PRO-M19	GP-M02	PGR-M01	PGR-F01
1	1	2	2	1	2	2	2	1	1
2	1	1	1	1	1	1	1	1	1
3	2	2	2	2	1	1	2	2	1
4	2	2	2	2	2	2	2	2	2
5	2	3	3	2	3	3	3	3	3
6	1	1	1	1	1	1	1	1	1
7	1	2	1	1	1	1	1	1	1
8	1	1	1	1	1	1	1	1	1
9	1	2	2	2	1	1	2	1	1
10	3	2	2	2	2	2	2	2	1
11	2	3	2	2	2	2	2	2	2
12	1	1	1	1	1	1	1	1	1
13	1	3	2	1	2	2	2	1	2
14	1	2	2	1	1	1	1	1	1
15	2	1	1	1	1	1	1	1	1
16	2	2	2	2	2	1	2	1	1
17	1	1	1	2	1	1	2	1	1
18	1	2	2	1	1	2	2	1	1
19	2	2	1	1	1	1	1	1	1
20	1	3	1	1	1	1	1	1	1
21	3	3	1	1	1	1	1	1	1
22	2	3	2	2	2	2	2	1	2
23	3	2	2	2	2	2	2	2	2
24	1	3	3	2	2	2	3	2	2
25	2	3	3	2	2	2	2	1	2
26	3	2	3	3	3	2	3	3	3
27	2	3	3	2	2	2	3	2	3
28	3	3	3	2	2	2	3	3	3
29	2	3	3	2	2	3	2	1	3
30	1	1	1	2	1	1	1	1	1
31	1	1	1	1	1	1	1	1	1
32	1	2	1	1	1	1	1	1	1
33	2	1	1	1	1	1	1	1	1
34	2	1	2	2	1	2	2	1	2
35	1	2	2	2	2	2	2	1	2
36	1	3	3	3	3	3	3	2	3
37	2	2	2	2	2	2	2	2	2
38	3	3	3	2	3	3	3	3	3
39	1	3	3	2	2	2	2	2	3
40	1	2	2	2	1	2	2	2	1
41	1	1	2	1	1	1	1	1	1
42	1	1	1	1	1	1	1	1	1
43	3	2	2	2	2	2	2	1	2
44	3	2	3	3	3	2	3	3	3
45	2	2	2	2	2	2	3	2	2
46	2	1	1	2	1	2	2	1	2
47	1	3	2	1	1	3	1	1	1
48	1	2	2	2	1	1	2	1	1
49	2	2	2	1	1	1	1	1	1
50	1	2	3	2	2	2	2	2	2
51	1	1	1	1	1	1	2	1	1
52	1	3	2	2	1	2	2	2	2
53	1	3	1	1	1	1	1	1	1
54	1	2	2	2	1	2	2	1	1
55	2	3	3	2	3	3	3	1	3
56	2	2	2	2	1	1	2	1	2
57	2	1	2	2	2	2	2	1	2
58	2	2	2	1	1	2	1	1	2
59	1	1	2	2	1	2	2	1	2
60	2	2	2	2	1	2	2	1	2
61	1	2	2	2	2	2	2	1	2
62	2	2	2	2	2	2	2	2	2
63	2	1	1	2	1	1	2	1	2
64	2	2	2	2	1	2	2	1	2
65	3	2	1	1	1	2	1	1	1
66	1	3	3	3	3	3	2	2	3
67	2	2	2	2	1	2	2	2	1
68	2	3	3	1	3	3	2	2	3
69	2	2	2	2	2	2	2	1	2
70	3	3	2	1	1	1	1	1	1
71	1	3	2	2	2	3	2	1	2
72	2	2	1	1	1	1	1	1	1
73	2	3	2	2	1	2	2	1	2
74	2	1	1	1	1	1	1	1	1
75	1	2	2	2	2	2	2	1	2
76	2	1	2	2	1	2	2	1	2
77	2	2	2	2	2	2	2	1	2
78	2	2	2	2	2	2	2	2	3
79	2	2	3	2	2	2	2	1	2
80	3	1	2	2	1	2	2	1	1
81	1	3	2	2	3	2	2	1	2
82	2	1	1	1	1	1	1	1	1
83	2	2	2	1	1	2	1	1	1
84	2	1	2	2	1	2	2	1	1
85	1	3	3	3	2	3	3	3	3
86	3	2	1	1	1	1	1	1	1
87	1	3	3	3	3	3	2	3	3
88	2	1	1	1	1	2	1	1	1
89	1	3	3	2	2	3	3	2	3
90	3	2	2	1	1	2	1	1	1
91	3	2	2	2	2	2	2	2	2
92	3	1	2	1	2	2	2	1	2
93	3	3	3	2	3	3	2	2	3
94	2	1	1	1	1	2	1	1	1
95	2	2	2	2	2	2	2	2	2
96	1	2	2	1	1	2	2	1	2
97	1	2	1	1	1	2	1	1	1
98	3	2	1	1	1	1	1	1	1
99	3	1	2	1	2	2	2	1	2
100	1	3	3	1	2	3	3	2	3
