# Supplementary material for: Reproducibility of Mandibular Cortical Index Classification Among Dental Examiners and a Single Generative AI Platform: An Observer Agreement Study
Source: Clin Pract. 2026 Jul 31;16(8):142. doi: 10.3390/clinpract16080142 (PMC13511330; doi:10.3390/clinpract16080142)
Supplement: Supplementary file 1 [file clinpract-16-00142-s001.zip › Supplementary_Table_S3_Pairwise_Kappa_Session1.pdf]

	AI-NBLM	END-M33	PER-M28	PRO-F29	PRO-M19	RAD-REF	GP-M02	PGR-M01	PGR-F01
**AI-NBLM**	-								
**END-M33**	0.053<br>[-0.101, 0.207]	-							
**PER-M28**	0.049<br>[-0.106, 0.204]	0.56<br>[0.412, 0.708]	-						
**PRO-F29**	0.022<br>[-0.119, 0.163]	0.437<br>[0.285, 0.589]	0.346<br>[0.198, 0.495]	-					
**PRO-M19**	0.089<br>[-0.063, 0.241]	0.491<br>[0.334, 0.649]	0.498<br>[0.344, 0.652]	0.539<br>[0.389, 0.689]	-				
**RAD-REF**	0.005<br>[-0.146, 0.156]	0.439<br>[0.287, 0.591]	0.308<br>[0.158, 0.457]	0.352<br>[0.201, 0.503]	0.51<br>[0.353, 0.667]	-			
**GP-M02**	0.128<br>[-0.038, 0.294]	0.397<br>[0.245, 0.549]	0.344<br>[0.198, 0.489]	0.623<br>[0.473, 0.773]	0.631<br>[0.485, 0.777]	0.438<br>[0.287, 0.589]	-		
**PGR-M01**	-0.012<br>[-0.165, 0.141]	0.264<br>[0.118, 0.410]	0.19<br>[0.054, 0.325]	0.455<br>[0.301, 0.609]	0.385<br>[0.155, 0.615]	0.401<br>[0.239, 0.563]	0.454<br>[0.302, 0.606]	-	
**PGR-F01**	-0.067<br>[-0.205, 0.071]	0.48<br>[0.324, 0.636]	0.453<br>[0.315, 0.591]	0.144<br>[-0.012, 0.300]	0.26<br>[0.114, 0.406]	0.227<br>[0.076, 0.378]	0.139<br>[-0.005, 0.283]	0.055<br>[-0.093, 0.203]	-
