# Supplementary material for: Reproducibility of Mandibular Cortical Index Classification Among Dental Examiners and a Single Generative AI Platform: An Observer Agreement Study
Source: Clin Pract. 2026 Jul 31;16(8):142. doi: 10.3390/clinpract16080142 (PMC13511330; doi:10.3390/clinpract16080142)
Supplement: Supplementary file 1 [file clinpract-16-00142-s001.zip › Supplementary_Table_S4_Pairwise_Kappa_Session2.pdf]

	AI-NBLM	END-M33	PER-M28	PRO-F29	PRO-M19	RAD-REF	GP-M02	PGR-M01	PGR-F01
**AI-NBLM**	-								
**END-M33**	0.003<br>[-0.158, 0.164]	-							
**PER-M28**	0.097<br>[-0.068, 0.261]	0.534<br>[0.385, 0.683]	-						
**PRO-F29**	0.129<br>[-0.036, 0.294]	0.205<br>[0.046, 0.364]	0.501<br>[0.344, 0.657]	-					
**PRO-M19**	0.091<br>[-0.073, 0.256]	0.436<br>[0.277, 0.595]	0.649<br>[0.508, 0.790]	0.444<br>[0.281, 0.607]	-				
**RAD-REF**	0.129<br>[-0.032, 0.290]	0.345<br>[0.198, 0.493]	0.52<br>[0.367, 0.673]	0.504<br>[0.351, 0.656]	0.55<br>[0.405, 0.695]	-			
**GP-M02**	0.097<br>[-0.068, 0.262]	0.314<br>[0.158, 0.470]	0.688<br>[0.557, 0.820]	0.671<br>[0.532, 0.810]	0.566<br>[0.417, 0.715]	0.532<br>[0.381, 0.682]	-		
**PGR-M01**	0.117<br>[-0.047, 0.282]	0.242<br>[0.101, 0.383]	0.341<br>[0.180, 0.501]	0.424<br>[0.261, 0.587]	0.315<br>[0.154, 0.475]	0.523<br>[0.368, 0.677]	0.4<br>[0.239, 0.561]	-	
**PGR-F01**	0.161<br>[0.000, 0.321]	0.382<br>[0.237, 0.527]	0.672<br>[0.533, 0.811]	0.562<br>[0.428, 0.697]	0.667<br>[0.528, 0.807]	0.682<br>[0.549, 0.815]	0.678<br>[0.545, 0.811]	0.422<br>[0.263, 0.581]	-
